# Metabolic reprogramming in triple-negative breast cancer

**DOI:** 10.20892/j.issn.2095-3941.2019.0210

**Published:** 2020-02-15

**Authors:** Zhanyu Wang, Qianjin Jiang, Chenfang Dong

**Affiliations:** ^1^Department of Surgical Oncology (Breast Center) of The Second Affiliated Hospital, Zhejiang University School of Medicine, Hangzhou 310058, China; ^2^Department of Pathology and Pathophysiology, Zhejiang University School of Medicine, Hangzhou 310058, China

**Keywords:** Metabolic reprogramming, triple-negative breast cancer, aerobic glycolysis, Warburg effect, cancer stem cell, targeted therapy

## Abstract

Since triple-negative breast cancer (TNBC) was first defined over a decade ago, increasing studies have focused on its genetic and molecular characteristics. Patients diagnosed with TNBC, compared to those diagnosed with other breast cancer subtypes, have relatively poor outcomes due to high tumor aggressiveness and lack of targeted treatment. Metabolic reprogramming, an emerging hallmark of cancer, is hijacked by TNBC to fulfill bioenergetic and biosynthetic demands; maintain the redox balance; and further promote oncogenic signaling, cell proliferation, and metastasis. Understanding the mechanisms of metabolic remodeling may guide the design of metabolic strategies for the effective intervention of TNBC. Here, we review the metabolic reprogramming of glycolysis, oxidative phosphorylation, amino acid metabolism, lipid metabolism, and other branched pathways in TNBC and explore opportunities for new biomarkers, imaging modalities, and metabolically targeted therapies.

## Introduction

Breast cancer is a heterogeneous disease comprising four major molecular subtypes, namely, luminal A, luminal B, human epidermal growth factor receptor 2 (HER2), and triple-negative^1^. Triple-negative breast cancer (TNBC) is defined as a tumor that lacks the expression of estrogen receptor (ER), progesterone receptor (PR), and HER2 (also known as ERBB2)^2^. Basal-like breast cancer (BLBC), which is a major subtype of breast cancer according to PAM50 subtyping, is generally categorized as the TNBC subtype. Patients with the TNBC subtype account for 15% of all breast cancer patients and have a relatively poor prognosis due to the high metastatic capacity of their tumors, especially to the brain and lungs^3^. The high risk of metastasis in these patients may be associated with epithelial-mesenchymal transition (EMT), which is induced by several transcriptional factors such as Snail1, Smad2, and Twist1/2. EMT confers TNBC with cancer stem cell (CSC)-like characteristics, including elevated metastatic potential and enhanced resistance to chemotherapy^4^. Nevertheless, chemotherapy currently remains the mainstay of systemic treatment due to the lack of effective targeted therapy such as anti-HER2 and anti-ER^5^. In addition, patients carrying a mutation in the breast cancer susceptibility gene (*BRCA1*) usually exhibit the triple-negative or basal-like phenotype and tend to be sensitive to the inhibitor of poly ADP-ribose polymerase (PARP)^3^. Although some patients with TNBC have a relatively better prognosis after chemotherapy or treatment with PARP inhibitors, many TNBC patients are still insensitive to these therapies. Hence, there is an urgent need to identify new druggable targets.

Cancer cells display significant metabolic reprogramming, which enables them to survive and rapidly proliferate in a nutrient-poor tumor microenvironment. For example, cancer cells preferentially utilize glycolysis despite the availability of adequate oxygen (known as aerobic glycolysis or the Warburg effect), thereby maintaining high levels of glycolytic intermediates for biosynthetic requirements, while decreasing the production of reactive oxygen species (ROS) from oxidative phosphorylation (OXPHOS)^6^. This rewired metabolic network depends on the dysregulation of many key enzymes, such as pyruvate kinase muscle isozyme M2 (PKM2), which redirects glycolytic intermediates into the anabolic pathway^7^. Appropriate intervention against the dysregulation of these pivotal enzymes may rectify cancer metabolism and hinder tumor growth, suggesting novel therapeutic strategies.

## Glycolysis in TNBC

### Aberrant expression of glycolysis-related enzymes

**Figure 1:**
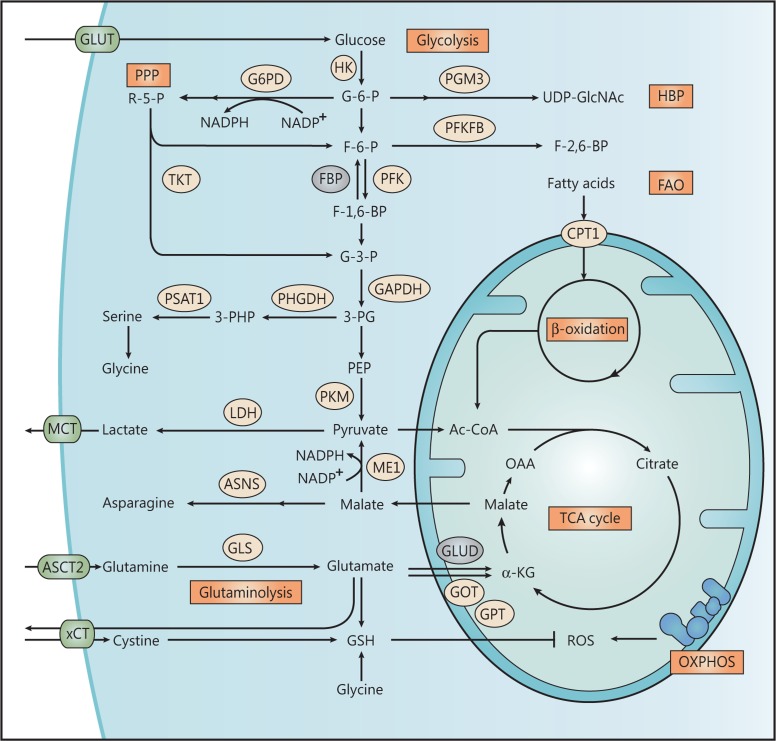
Metabolic reprogramming in triple-negative breast cancer (TNBC). TNBC upregulates several key glycolytic enzymes and transporters such as GLUT, HK, LDH, and MCT, therefore displaying high rate of glycolysis and glycolytic branched pathways, including serine synthesis and pentose phosphate pathway (PPP) for NADPH generation, and hexosamine biosynthetic pathway (HBP) for protein glycosylation. Other pathways such as fatty acid oxidation (FAO), glutaminolysis and cystine uptake are also induced in TNBC to meet its bioenergetic or biosynthetic demands, and mitigate reactive oxygen species (ROS) that is generated from oxidative phosphorylation (OXPHOS). Enzymes that are upregulated in TNBC are shown as light-yellow ovals, and enzymes downregulated in TNBC are shown as gray ovals.

Compared with other breast cancer subtypes, TNBC is more dependent on glycolysis; it displays elevated glucose uptake and lactate secretion, with the upregulation of several key glycolytic enzymes and transporters, including lactate dehydrogenase (LDH), glucose transporter (GLUT), and monocarboxylate transporter (MCT) (**Figure 1**). Knocking down GLUT4 diminishes glucose uptake and lactate release, thus normalizing the metabolism of cancer by reallocating the glycolytic flux to OXPHOS, which leads to compromised cell proliferation and viability under hypoxia^8^. Either tumoral LDH-A or LDH-B is closely associated with poor clinical outcomes in patients with TNBC^9,10^. Two isoforms of MCT, MCT1 and MCT4, are also specifically upregulated in TNBC, where they mediate lactate extrusion and acidification of the tumor microenvironment^9,11^. Disruption of lactate transport impairs tumor growth *in vitro* and *in vivo*, accompanied by reduced tumor aggressiveness^12^.

### Crosstalk between glycolysis and oncogenic signaling

**Figure 2:**
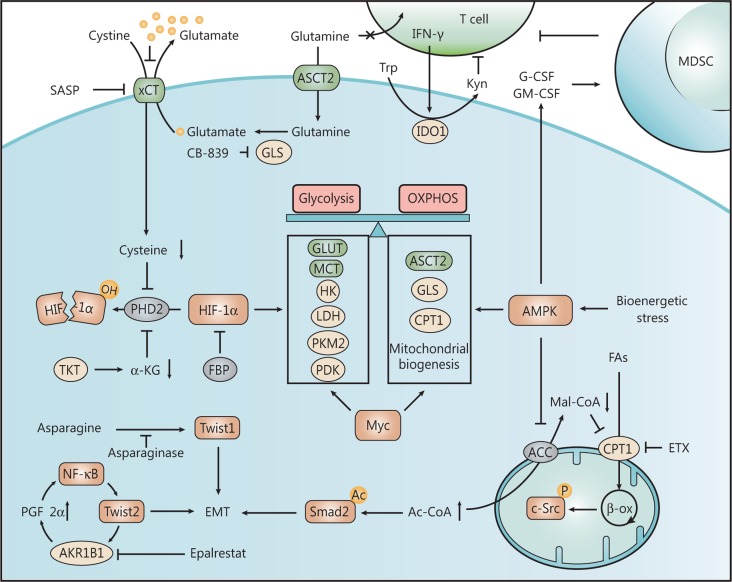
Switchable metabolic dependence on glycolysis and oxidative phosphorylation (OXPHOS) regulated by HIF-1α and AMPK. Under normoxic condition, HIF-1α is hydroxylated and subsequently degraded by PHD2 in an α-KG- and cysteine-dependent manner. In TNBC, decreased levels of α-KG and cysteine due to altered activity of transketolase (TKT) and xCT cystine/glutamate antiporter, respectively, contribute to normoxic activation of HIF-1α signaling, which triggers aerobic glycolysis with the upregulation of glycolysis-related enzymes and transporters. Although TNBC cells rely on glycolysis for rapid proliferation, OXPHOS which generate ATP more efficiently is also required, especially under bioenergetic stress. Activated AMPK and Myc pathways induce mitochondrial biogenesis and enzymes involved in glutaminolysis and fatty acid oxidation (FAO) to facilitate OXPHOS, thus endowing TNBC with metabolic plasticity to switch between glycolysis and OXPHOS. In addition, AMPK signaling also promotes epithelial-mesenchymal transition (EMT) through regulating lipid metabolism and increases the secretion of cytokines to recruit immunosuppressive myeloid-derived suppressor cells (MDSCs). Many drugs have been developed to target these key processes in the metabolic reprogramming of TNBC.

The aberrant expression of glycolysis-related enzymes in TNBC can be attributed to dysregulated signaling, such as the epidermal growth factor receptor (EGFR), HIF-1α, and c-Myc pathways. The EGFR signaling, frequently active in TNBC, triggers the expression of hexokinase 2 (HK2) and stabilizes GLUT1 on the cell membrane^13^, whereas it reduces the activity of PKM2^14^, thus causing an accumulation of the glycolytic intermediate, fructose-1,6-bisphosphate (F-1,6-BP). In turn, elevated F-1,6-BP directly binds to EGFR and enhances its activity, further promoting EGFR-mediated aerobic glycolysis. Combining the glycolytic inhibitor 2-deoxy-D-glucose with the EGFR inhibitor gefitinib effectively suppresses TNBC cell proliferation^14^. Under normoxic conditions, HIF-1α is hydroxylated by the proline hydroxylase domain (PHD2; also known as EglN1) in an oxygen- and α-ketoglutarate (α-KG)-dependent manner, which results in its ubiquitination and subsequent degradation^15^. However, a long non-coding RNA abundant in TNBC, LINK-A, can activate normoxic HIF-1α signaling. Mechanistically, LINK-A induces BRK-mediated phosphorylation of HIF-1α at Tyr 565, which interferes with the hydroxylation of the adjacent Pro 564 by PHD2, leading to normoxic HIF-1α stabilization^16^. Once activated, HIF-1α transcriptionally promotes the expression of most glycolytic enzymes and transporters (e.g., GLUT1, HK2, LDH-A, and MCT4) to enhance the ability of cancer cells to perform glycolysis. HIF-1α also inactivates the pyruvate dehydrogenase complex and attenuates the entry of pyruvate into the tricarboxylic acid (TCA) cycle, thereby reinforcing the glycolytic phenotype and decreasing OXPHOS^15,17^. BLBC with c-Myc activation overexpresses many c-Myc-targeted genes, including LDH-A, pyruvate dehydrogenase kinase (PDK), and glutaminase (GLS)^18^ (**Figure 2**). c-Myc also represses the level of thioredoxin-interacting protein, a potent negative regulator of glucose uptake and glycolytic gene expression, to further activate aerobic glycolysis^19^.

The metabolic rewiring of glycolysis due to dysregulated signaling can, in turn, promote oncogenic pathways through altered metabolites or energy status. Overexpression of GLUT3 in nonmalignant human breast cells contributes to loss of polarity and activates several known oncogenic pathways, including the EGFR, β1-integrin, MEK, and AKT pathways. In contrast, decreasing glucose uptake in breast cancer cells suppresses these oncogenic pathways and promotes the formation of organized structures^20^. In TNBC, glutathione S-transferase Pi 1 (GSTP1) interacts with and activates glyceraldehyde-3-phosphate dehydrogenase (GAPDH) to facilitate glycolytic flux, whereas the inhibition of GSTP1 by LAS17 impairs glycolysis and lowers the generation of ATP and macromolecular building blocks, such as lipids and nucleotides. This energy reduction ultimately blocks oncogenic signaling through the activation of AMPK and the inhibition of mTOR signaling^21^. Unexpectedly, the bifunctional enzyme 6-phosphofructo-2-kinase/fructose-2,6-bisphosphatase 4 (PFKFB4), a glycolytic stimulator responsible for the phosphorylation of F-6-P to F-2,6-BP, can also act as a protein kinase. PFKFB4 phosphorylates oncogenic steroid receptor coactivator-3 (Src-3) and enhances its transcriptional activity, which leads to the upregulation of transketolase (TKT), a major enzyme that mediates the non-oxidative pentose phosphate pathway (PPP), thus shunting glycolytic flux into the PPP for purine synthesis^22^. Overexpression of TKT in TNBC is also required for the activation of HIF-1α signaling. TKT depletion upregulates the expression of succinate dehydrogenase and fumarate hydratase, thereby decreasing the levels of oncometabolites, succinate and fumarate, both of which can suppress PHD2 activity and stabilize HIF-1α. Suppressing TKT by oxythiamine leads to the disruption of HIF-1α-induced aerobic glycolysis and breast cancer metastasis^23^. In addition, a high rate of aerobic glycolysis in TNBC increases the secretion of G-CSF and GM-CSF through the AMPK-ULK1-autophagy pathway, which promotes myeloid-derived suppressor cells (MDSCs) development and an immunosuppressive microenvironment^24^ (**Figure 2**).

## Amino acid metabolism

### Glutamine addiction and dysregulated glutaminolysis

Circulating glutamine is the most abundant amino acid in the blood. Although it is a non-essential amino acid, many cancers, including TNBC, are dependent on glutamine and hijack glutaminolysis, the process by which glutamine is catabolized for entry into the TCA cycle, to support biosynthesis, energy generation, and glutathione (GSH) production^25,26^. Under physiologic conditions, glutamine is transported into cells *via* many transporters, such as alanine, serine, cysteine-preferring transporter 2 (ASCT2; also known as SLC1A5), and L-type amino acid transporter 1 (LAT1; also known as SLC7A5). Intracellular glutamine is deaminated by GLS to glutamate, which can then be converted to α-KG by either glutamate dehydrogenase (GLUD) or several aminotransferases, such as glutamate-oxaloacetate transaminase (GOT), glutamate-pyruvate transaminase (GPT), and phosphoserine aminotransferase (PSAT), following which α-KG enters the TCA cycle^26^ (**Figure 1**).

In TNBC, both ASCT2 and LAT1 are overexpressed^27,28^. High expression of ASCT2 is critical for the uptake of glutamine and subsequent glutaminolysis, leading to the activation of the mTORC1 nutrient-sensing pathway^27^. Metabolomics analysis also reveals a low level of glutamine and a high level of glutamate in TNBC, indicating enhanced glutaminolysis^29^. Compared with other breast cancer subtypes, TNBC is more glutamine dependent and susceptible to glutaminolysis-targeting therapeutics because of the overexpression of GLS^30,31^, which is associated with high-grade metastatic breast cancer^32^. Several small-molecule inhibitors of GLS, such as CB-839, BPTES, and compound 968^33^, have been developed to target dysregulated glutaminolysis. In addition, GLS expression in TNBC is significantly correlated with a low level of tumor-infiltrating lymphocytes (TILs), suggesting a metabolic competition between cancer cells and TILs in the tumor microenvironment, where active consumption of intercellular glutamine by GLS-overexpressing TNBC cells deprives TILs of glutamine and hinders their proliferation^34^ (**Figure 2**). As explained above, glutamate is converted to α-KG through two mechanisms, *via* transaminases or GLUD. Compared with quiescent cells, highly proliferative cells prefer to catabolize glutamate *via* transaminases to synthesize non-essential amino acids (aspartate and alanine) and downregulate GLUD to reduce ammonia production. Consistently, among the four major breast cancer subtypes, the most proliferative basal breast tumors express high levels of GPT2 and PSAT1, whereas they express relatively low levels of GLUD1/2^35^. In contrast, ER-positive breast cancers exhibit increased GLUD expression, which accounts for their glutamine independence. Mechanistically, GLUD reversibly catalyzes the reductive amination of α-KG to glutamate under glutamine deprivation. Through this metabolic recycling of ammonia, elevated glutamate levels enable the synthesis of other amino acids, such as aspartate and proline^36^. Another reason for the glutamine independence of luminal-type breast cancer is the high expression of glutamine synthetase (GS), which is directly induced by a key luminal transcription factor, GATA3. Luminal cells can rescue basal cells in co-culture without glutamine, indicating possible glutamine symbiosis within breast ducts^37^. In addition to GPT2 and PSAT1, another transaminase, GOT2, is also overexpressed in TNBC; it facilitates cell proliferation by increasing aspartate and α-KG production. BRCA1 protein transcriptionally represses GOT2 expression, but this repression mechanism is impaired due to the frequently observed *BRCA1* deficiency in TNBC^3,38^. Intriguingly, as the product of glutamate, gamma-aminobutyric acid (GABA), is a major neurotransmitter in mammals, the catabolic pathway of GABA is remodeled. GABA is catabolized to succinic semialdehyde by gamma-aminobutyrate aminotransferase (ABAT). We discovered that, compared with other subtypes, ABAT is considerably decreased in BLBC due to Snail-mediated transcriptional repression, thus causing the accumulation of GABA; the elevated GABA then activates GABA-A receptor (GABAA) and subsequently triggers the activation of Ca^2+^-NFAT1 signaling to promote the aggressive behavior of BLBC. In breast tumor patients, loss of ABAT is strongly correlated with large tumor size, high tumor grade, and metastatic tendency^39^.

### Cystine uptake by xCT is required for the CSC phenotype

High levels of glutaminolytic flux and glutamate indirectly support environmental cystine acquisition *via* the xCT cystine/glutamate antiporter (SLC7A11), a major transporter for the uptake of cystine in exchange for intracellular glutamate. The xCT antiporter is overexpressed in one-third of TNBCs and is essential for GSH synthesis and the maintenance of CSCs^31^. Silencing of xCT impairs tumorsphere formation and the redox balance in breast cancer stem cells (BCSCs)^31,40^. In turn, chemotherapy induces the enrichment of BCSCs in TNBC by upregulating xCT in a HIF-1-dependent manner to facilitate the synthesis of GSH and activate the gene encoding the pluripotency factor, Nanog^41^. The CD44 variant (CD44v), a marker of CSCs, interacts with and stabilizes xCT at the cell membrane^42^. Meanwhile, mucin 1 (MUC1), a transmembrane glycoprotein that is aberrantly overexpressed in TNBC, binds directly to the intracellular domain of CD44v and further promotes the stability of xCT^43^. Nevertheless, the accumulation of extracellular glutamate secreted by the xCT antiporter in turn inhibits the xCT antiporter and cystine uptake. Subsequently, the depletion of intracellular cysteine disables PHD2, which hydroxylates HIF-1α for degradation, thus leading to the induction of HIF-1α signaling and triple-negative breast carcinogenesis^44^ (**Figure 2**). The secreted glutamate can also induce metabotropic glutamate receptors (mGluR) on the membrane of TNBC and endothelial cells, promoting tumor growth and angiogenesis and inhibiting inflammation through the mGluR1 signaling. These tumor-promoting effects can be blocked by riluzole, a Food and Drug Administration (FDA)-approved drug for the treatment of amyotrophic lateral sclerosis^45–47^. Moreover, cystine starvation induces mitochondrial fragmentation and ROS production, leading to necroptosis and ferroptosis in BLBC cells *via* the tumor necrosis factor alpha (TNFα) and MEKK4-p38-Noxa pathways, while luminal-type breast cancer, without activation of these pathways, is cystine independent^48,49^.

### Serine synthesis promotes NADPH generation and anaplerosis

The serine synthetic pathway diverts glycolytic carbon fluxes from 3-phosphoglycerate into *de novo* serine and glycine biosynthesis, conferring several metabolic advantages. These advantages include limiting ATP production, synthesis of serine for one-carbon metabolism and NADPH formation, and the generation of α-KG from glutamate. Phosphoglycerate dehydrogenase (PHGDH), as a key enzyme in the first step of serine synthesis, is overexpressed in both TNBC and BLBC, in part, because of copy number amplification^50,51^. Ectopic expression of PHGDH in breast epithelial cells disrupts acinar morphogenesis and induces phenotypic alterations that may contribute to oncogenesis^52^. Under hypoxia or doxorubicin treatment, the expression of PHGDH and downstream enzymes in the serine synthesis or one-carbon cycle is upregulated. This metabolic reprogramming maintains the level of NADPH to counter hypoxia- or chemotherapy-induced ROS stress and plays a role in BCSC enrichment and lung metastasis^53,54^. Suppression of PHGDH leads to markedly decreased cell proliferation and serine synthesis; although it does not affect intracellular serine concentration, it lowers the level of α-KG, an intermediate in the TCA cycle that is produced by PSAT1, which is downstream of PHGDH. In cells with high PHGDH expression, the serine synthesis pathway shunts 8%–9% of the glycolytic flux towards serine production and simultaneously contributes approximately 50% of the total anaplerotic flux of glutamine into the TCA cycle as α-KG^50^. The overexpression of PHGDH and PSAT1 is significantly associated with poor clinical outcome and malignant phenotypic features of breast cancer^55^.

### Catabolism of tryptophan, arginine, and other amino acids

Most tryptophan catabolism occurs *via* the kynurenine pathway, which is catalyzed in the first step by indoleamine 2,3-dioxygenase (IDO) or tryptophan 2,3-dioxygenase (TDO)^56^. Tryptophan can also be catabolized to 5-hydroxytryptamine (5-HT; also known as serotonin) by tryptophan hydroxylase 1 (TPH1). The expression of these enzymes is aberrant in TNBC, and their products, kynurenine and 5-HT, may modulate immune surveillance or oncogenic signaling. For example, in response to T cell-derived interferon (IFN)-γ, TNBC exhibits highly inducible expression of IDO1 to counteract immune cells^57^ (**Figure 2**). Overexpression of TDO2 is also found in TNBC cells in suspension in a nuclear factor kappa B (NF-κB)-dependent manner, rendering TNBC more resistant to anoikis, a programmed cell death process triggered by substratum detachment during metastasis. Mechanistically, the increased kynurenine generated by TDO2 activates the aryl hydrocarbon receptor (AhR), an endogenous kynurenine receptor, thus facilitating the proliferation, invasion, and metastatic capacity of TNBC^58,59^. In addition, 5-HT promotes the invasion and proliferation of TNBC cells *via* 5-HT7 receptor and increases the expression of TPH1 and vascular endothelial growth factor (VEGF)^56^.

Arginine is required for the growth of TNBC because of its two products, ornithine and nitric oxide (NO). Arginine uptake and ornithine synthesis are induced during the S/G2/M phases in transformed cells but not in normal cells. Cancer cells exclusively use arginase 2 (ARG2) to synthesize ornithine, whereas normal epithelial cells depend on ornithine aminotransferase (OAT). Knockdown of ARG2 in BLBC markedly reduces cancer cell growth and causes G2/M arrest but does not prompt compensation *via* OAT^60^. Rosuvastatin may impede breast cancer development because it inhibits arginase enzymatic activity and reduces the levels of ornithine and polyamine^61^. Silencing of argininosuccinate lyase (ASL), an enzyme responsible for the production of arginine, also delays the G2/M transition of TNBC cell line^62^. Another use of arginine is to synthesize NO *via* nitric oxide synthase (NOS). A high activity of inducible NOS (iNOS) is associated with poor survival of TNBC patients. Increased generation of NO induces the EGFR pathway *via* S-nitrosylation and subsequently activates several oncogenic signal transduction pathways (including c-Myc, Akt, and β-catenin). The NO signaling also triggers upregulation of the stem cell marker CD44 and other proteins that are characteristic of BLBC, potentiating EMT, chemoresistance, and invasion capacity^63–66^. In addition, NO can directly inhibit the catalytic activity of the demethylase KDM3A; thereby, it alters cellular histone methylation patterns^67^, leading to changes in the expression levels of numerous oncogenes such as Ets-1^68^.

The branched-chain amino acid transaminase 1 (BCAT1), which is involved in the breakdown of branched-chain amino acids, is overexpressed in TNBC because of the hypomethylation on its promoter. BCAT1 indirectly controls cell cycle and enhances invasion capacity^69^. Asparagine, methionine, and glutamine metabolism are also essential for modulating the metastasis, stemness, and apoptosis of TNBC. Dietary restriction or otherwise inhibiting the synthesis of these amino acids has tumor-suppressing effects, which is discussed later in the section of clinical practice^70–73^.

## Lipid metabolism

### Increased oxidation and decreased synthesis of fatty acid

In addition to glucose and amino acids, cancer cells also utilize fatty acids (acquired from the extracellular matrix or from *de novo* synthesis) as an alternative energy source through fatty acid oxidation (FAO), which is a very efficient way to produce energy. FAO begins with the acylation of fatty acid to form acyl-CoA, and then, acyl-CoA is transported into the mitochondria through carnitine palmitoyltransferase (CPT) as the first committed step of the FAO. Myc-overexpressing TNBC shows upregulated CPT activity and increased bioenergetic reliance on FAO through targeted metabolomics analysis^74^. Upregulation of CPT1C promotes FAO and ATP generation, contributing to cell resistance against metabolic stress such as hypoxia, glucose deprivation, or mTOR inhibition^75^. Increased levels of ATP through mitochondrial FAO also activate the oncoprotein Src *via* autophosphorylation, whereas interfering with CPT1 abolishes Src activation and reverses the Src-regulated gene pattern, leading to decreased tumor growth and metastasis *in vivo*^76^. Elevated FAO in TNBC is associated with the upregulation of peroxisome proliferator-activated receptor gamma coactivator 1-alpha (PGC1-α), the master regulator of mitochondria biogenesis and respiration^74^, which activates FAO to promote energy homeostasis and cell viability, especially under attachment loss or metabolic stress, thus promoting the metastasis of TNBC^77^. In contrast, fatty acid synthesis (FAS) and lipogenic enzymes are downregulated in TNBC compared with the HER2 subtype [upregulating ATP citrate lyase (ACLY) and fatty acid synthase (FASN) to accelerate FAS]^25,74^. Acetyl-CoA carboxylase (ACC) is the key lipogenic enzyme that irreversibly catalyzes acetyl-CoA to malonyl-CoA, which is an inhibitor of CPT1 and FAO. TNBC downregulates ACC activity *via* post-translational modification to decrease malonyl-CoA generation and boost FAO flux. Mechanistically, PHD3 hydroxylates ACC2 to promote its activity, whereas loss of PHD3 in BCSCs inactivates ACC2 and enables tumor metabolic reliance on FAO^78,79^. In addition, in response to TGF-β or leptin, ACC1 is phosphorylated and inactivated through the AMPK signaling, resulting in the elevation of cellular acetyl-CoA, which promotes the acetylation of EMT-inducing Smad2 and, ultimately, EMT programs and metastasis^80^ (**Figure 2**). Nevertheless, blocking FASN and lipogenesis by metformin or EGCG also induces antitumor effects on TNBC^81,82^, suggesting an intricate role of FAS in cancer cells.

### Other lipid metabolic pathways

Triglycerides in circulating lipoprotein particles can provide an additional, exogenous source of fatty acid. TNBC exhibits enhanced lipid uptake by secreting lipoprotein lipase (LPL, which hydrolyzes the triglycerides in lipoproteins into fatty acids) and expressing CD36 (the channel for cellular fatty acid uptake)^83^. Several fatty acid-binding proteins, including FABP5 and FABP7, have also been identified as prognostic markers for poor outcomes in TNBC. They promote cell proliferation by enhancing fatty acid uptake and activating the retinoic acid pathway^84,85^. Additionally, cholesterol biosynthesis is enhanced through the upregulation of HMG-CoA reductase (HMGCR) and HMG-CoA synthase 1 (HMGCS1), key enzymes in the mevalonate pathway^86^. Meanwhile, elevated activity of low-density lipoprotein (LDL), caveolin-1, and cholesterol acyltransferase 1 (ACAT1) in TNBC is associated with greater uptake and utilization of cholesterol^87^. We have reported that the aldo-keto reductase 1 member B1 (AKR1B1) is activated in TNBC by Twist2, an inducer of EMT, and the accumulation of its major metabolite PGF2α, in turn, activates NF-κB signaling to upregulate Twist2 expression, which eventually forms a positive feedback loop to enhance the CSC properties in TNBC^88^ (**Figure 2**). Lipin-1 is a phosphatidic acid phosphatase that controls the synthesis of phospholipid for membrane biogenesis. In TNBC, lipin-1 is dramatically upregulated, whereas knockdown of lipin-1 blocks phospholipid synthesis and changes the membrane lipid composition, leading to apoptosis and growth arrest^89^.

## OXPHOS, ROS, and the anti-oxidant pathway

### OXPHOS is downregulated to reduce ROS production

Although OXPHOS generates more ATP than glycolysis does, when oxygen availability is compromised, electrons can escape from the electron transport chain, and they can be captured by O2, causing excessive ROS (such as superoxide and hydrogen peroxide) and ROS-mediated DNA damage^90,91^. Hence, most tumors, including TNBC, tend to utilize glycolysis to meet bioenergetic demands and rigidly control the level of ROS by downregulating OXPHOS. Consistently, metabolomics analysis of TNBC cell lines cultured under hypoxia shows that these cells have increased glucose uptake and higher glycolytic rate, while the conversion rate of glucose into the TCA cycle for OXPHOS is decreased^92^. We have discovered that a key gluconeogenic enzyme, fructose-1,6-bisphosphatase (FBPase), is a key regulator that triggers the metabolic switch from glycolysis to OXPHOS. Ubiquitous loss of FBP1 in TNBC is required for the maintenance of aerobic glycolysis and the CSC phenotype^91,93^. Mechanistically, FBP1 suppresses HIF-1α activity by directly binding to its inhibitory domain, thus acting as a transcriptional corepressor in the nucleus to repress the transcription of HIF-1α target glycolysis-related genes, including GLUT1, LDHA, and PDK1^94^. FBP1 also enhances OXPHOS by activating mitochondrial electron transporter complex I through the upregulation of the mitochondrial transcription factor B1M (TFB1M), which is essential for complex I protein translation^93^. Nevertheless, it has been reported that Myc and MCL1 cooperatively promote OXPHOS and ROS generation to activate HIF-1α signaling, which confers chemoresistance by expanding BCSCs^95^. A similar mechanism is observed in TNBC cells with mitophagy defect^96^. The roles of ROS and OXPHOS in TNBC remain controversial and require further exploration.

### Powerful anti-oxidant system

As described, through the high GLS1 activity and enhanced uptake of cystine, TNBC cells can quickly synthesize abundant GSH to antagonize transitory ROS elevation. To accelerate the regeneration of reduced GSH, some metabolic pathways relevant to NADPH formation are also upregulated in TNBC. We found that malic enzyme 1 (ME1), a cytosolic NADP-dependent enzyme that decarboxylates malate to pyruvate with NADPH generation, is dramatically upregulated in BLBC, where it serves as part of an important NADPH-supplying pathway^97^ (**Figure 1**). Nicotinamide phosphoribosyltransferase (NAMPT) is a rate-limiting enzyme in the synthesis of NAD^+^. TNBC cells utilize NAMPT to increase the intracellular NAD^+^ pool, which is converted to NADP^+^ and finally reduced to NADPH *via* the PPP^98^. In addition, ferritin is a family of proteins associated with iron storage, oxidation, and deposition. Some TNBC cell lines show aberrantly increased heavy subunits of ferritin in the nucleus, where it can act as a GSH-independent anti-oxidative system, protecting the DNA from damage by oxidizing Fe^2+^ to Fe^3+^ to mitigate ROS stress after exposure to UV^99,100^.

### Switchable dependence on glycolysis or OXPHOS

Although TNBC relies on metabolic reprogramming from OXPHOS to glycolysis, and glycolytic inhibitor can indeed significantly suppress the aggressiveness and CSC phenotype of TNBC cells^101^, it is noteworthy that inhibition of glycolysis also promotes the transition of BCSC in TNBC from a quiescent, mesenchymal-like state [characterized by high expression of aldehyde dehydrogenase (ALDH)] to a proliferative, epithelial-like state (characterized by CD24^-^CD44^+^ expression) through ROS-induced AMPK-HIF-1α pathway, in a process that resembles the EMT process. Epithelial-like BCSCs exhibit elevated OXPHOS coupled with the upregulation of GSH metabolism and anti-oxidant defense^102^, which is also observed in “energetic” cancer stem cells (e-CSCs) and TNBC brain or lung metastases^103–106^. Similarly, after chemotherapy, the residual tumors are highly sensitive to the inhibitor of OXPHOS, suggesting increased dependence on OXPHOS^107^. In addition, loss of the tumor-suppressor retinoblastoma gene (*RB1*) in TNBC induces mitochondrial protein translation, OXPHOS, and anabolic metabolism, thus equipping cancer cells to utilize scant oxygen levels and migrate away from the hypoxic area^108,109^. In fact, TNBC cells stably maintain a hybrid metabolic phenotype that is characterized by both high activity of glycolysis/OXPHOS and high levels of HIF-1/AMPK. Cells with this metabolic phenotype display maximum proliferation and clonogenicity relative to cells exhibiting a more glycolytic or more OXPHOS phenotype. The hybrid metabolic phenotype endows TNBC with the metabolic plasticity to switch between glycolysis and OXPHOS as a compensatory strategy in response to metabolic targeting drugs or an altered tumor environment^110^ (**Figure 2**). Therefore, dual targeting of glycolysis and mitochondrial bioenergetics or antioxidant pathways decreases cellular bioenergetics and increases the death of breast cancer cells^102,111^. Understanding this switchable metabolic dependence mechanism during tumor development and clinical process will facilitate the development of precise treatments.

## Other metabolic pathways

Recently, we reported that increased expression of urine diphosphate–galactose ceramide galactosyltransferase (UGT8) in BLBC facilitates tumor aggressiveness through the sulfatide-αVβ5 axis and predicts poor prognosis. Mechanistically, UGT8 is transcriptionally upregulated by Sox10 to induce the biosynthesis of sulfatide, which then activates integrin αVβ5-mediated signaling, including TGF-β and NF-κB, to potentiate tumor viability and metastasis^112^. Dysregulation of protein glycosylation, resulting from an increased flux of the hexosamine biosynthetic pathway (HBP), contributes to breast tumor progression or metastasis^113^. N-acetylglucosamine-phosphate mutase (PGM3), which is responsible for the conversion of N-acetylglucosamine-6-P (GlcNAc-6-P) into N-acetylglucosamine-1-P (GlcNAc-1-P), is the key enzyme in the synthesis of UDP-GlcNAc, the donor substrate of protein glycosylation. In TNBC, the inhibition of PGM3 blocks the HBP and alters glycosylation levels and the stability of proteins, leading to unfolded protein response and arrested cell proliferation^114^. The *de novo* synthesis of pyrimidine from glutamine is induced in TNBC subjected to chemotherapy. Metabolic flux through this pathway is controlled by the multifunctional enzyme carbamoyl-phosphate synthetase 2, aspartate transcarbamoylase, dihydroorotase (CAD), and dihydroorotate dehydrogenase (DHODH). Exposure to doxorubicin likely stimulates pyrimidine synthesis by regulating the post-translational modification of CAD and its activity, whereas inhibiting DHODH sensitizes TNBC cells to doxorubicin and significantly induces the regression of TNBC xenografts^115^. In addition, targeting DHODH causes synthetic lethality in phosphatase and tensin homolog (PTEN)-mutant TNBC due to inherent defects in DNA repair, leading to accumulated DNA damage and impaired cell replication^116^.

## Metabolic reprogramming of TNBC in the clinic

### Functional imaging

Neo-adjuvant chemotherapy based on anthracyclines and taxanes is increasingly used prior to surgery to reduce the size of unresectable TNBC. Traditionally, the response to chemotherapy is assessed by tumor volume, which represents a post-treatment event^117^. Because of the significantly higher uptake of glucose and the glucose analogue 18F-fluorodeoxyglucose (18F-FDG), 18F-FDG PET/CT is used for early determination of TNBC sensitivity to neo-adjuvant chemotherapy and for prediction of recurrence after surgery^118,119^. Additionally, the thymidine analogue 3’-[18F]fluoro-3’-deoxythymidine (18F-FLT), which reflects the activity of thymidine kinase and pyrimidine salvage, is also a potential marker of chemotherapy efficacy in TNBC^117^. By using high-resolution 1H magnetic resonance spectroscopy (MRS), TNBC with high GLS activity exhibits low cellular glutamine pool size (glutamine concentration), which significantly increases after the inhibition of GLS^120^. Recently, novel imaging techniques have been developed, such as [18F](2S,4R)4-fluoroglutamine (18F-FGln) PET and glutamate-weighted chemical exchange saturation transfer MR imaging (GluCEST MRI), to study glutamine transport and kinetics. Similar to MRS, these methods enable the observation of altered levels of glutamine or glutamate upon GLS inhibition, indicating their feasibility to non-invasively detect the early therapeutic response of TNBC to GLS inhibitors^120,121^.

### Targeting glutaminolysis and cystine uptake

Several classes of compounds have been developed to target GLS and block glutaminolysis in TNBC, and among these compounds, the most promising is CB-839, a potent, selective, and orally bioavailable inhibitor of GLS. CB-839 has an antiproliferative effect on TNBC cells both *in vitro* and in xenograft models, accompanied by marked decreases in glutaminolytic flux, oxygen consumption, and levels of GSH and TCA cycle intermediates^122^. In clinical trials, CB-839 (Telaglenastat) has been shown to be safe, and it exhibits a disease control rate of 55% in combination with paclitaxel in TNBC patients who are refractory to taxane therapy. CB-839 also suppresses mTOR activity in TNBC and synergizes with mTOR inhibition^30^. In combination with the mTOR inhibitor everolimus, it shows a disease control rate of 92% in renal cell carcinoma patients; the FDA has granted fast track designation for CB-839 in combination with everolimus or cabozantinib for the treatment of renal cell carcinoma^123^. Other combination therapies, such as the combination of GLS inhibition and bevacizumab (an anti-angiogenesis monoclonal antibody that targets VEGF), also exhibit antitumor effects on TNBC^124^. In addition, increased generation of glutamate by GLS can support the uptake of exogenous cystine through the cystine/glutamate antiporter xCT to maintain the redox balance. Sulfasalazine (SASP), as a clinically approved anti-inflammatory drug, is found to inhibit xCT activity and retards TNBC growth^31^ (**Figure 2**). By immunizing mice with a DNA-based vaccine expressing xCT protein, immunotargeting of the xCT antigen on the cell surface efficiently attenuates tumor growth and pulmonary metastasis and increases BCSC chemosensitivity to doxorubicin^40^. However, because the antibody titers achieved upon using the DNA vaccine are low, a virus-like-particle (VLP; AX09-0M6) immunotherapy has been developed, which elicits a stronger antibody response against xCT to decrease BCSC growth and prevent self-renewal^125^.

### Dietary restriction of amino acids

Besides glutamine and cysteine, TNBC cells are also dependent, to some extent, on the availability of certain other amino acids such as methionine, asparagine, and arginine, which suggests that limiting the supply of these amino acids may confer therapeutic benefits. The depletion of either methionine or glutamine can increase the cell surface expression of pro-apoptotic TNF-related apoptosis-inducing ligand receptor-2 (TRAIL-R2) and sensitize TNBC cells to TRAIL-induced apoptosis^71,72^. Dietary methionine deprivation enhances cell susceptibility to lexatumumab, an agonistic monoclonal antibody targeting TRAIL-R2, and reduces the lung metastasis rate^71,73^. Additionally, many tumor and stem cells depend on the biosynthesis of the universal methyl-donor S-adenosylmethionine (SAM) from exogenous methionine by methionine adenosyltransferase 2α (MAT2A) to maintain their epigenome^126,127^. Methionine restriction is sufficient to cripple the tumor-initiating capability of TNBC partly because of impaired SAM generation. Limiting dietary methionine induces MAT2A expression in TNBC as an adaptive response; therefore, the combination of methionine restriction and the MAT2A inhibitor cycloleucine has a synergistic antitumor effect^128^. Under normal physiological conditions, the asparagine levels in the serum are lower than those in the mammary gland, thus making asparagine bioavailability a key regulator of circulating tumor cells and the metastatic potential of breast cancer. Limiting asparagine by knocking down asparagine synthetase (ASNS), treatment with l-asparaginase, or dietary asparagine restriction suppresses metastasis and decreases the level of asparagine-enriched proteins, especially proteins associated with EMT, which, at least in part, accounts for the inhibition of metastasis progression^70^ (**Figure 2**). The depletion of arginine by recombinant human arginase (rhArg) leads to TNBC cell apoptosis *via* ROS and induces adaptive autophagy, whereas blocking autophagic flux by autophagy-targeting drugs potentiates the cytotoxicity of rhArg^129^. Other dietary modifications, such as caloric restriction, also cause tumor regression in combination with radiation^130^.

### Blocking other metabolic pathways

Overexpression of Myc induces a bioenergetic reliance on FAO in TNBC cells, especially under glucose deprivation or matrix detachment. Etomoxir (ETX), as a clinically tested, specific inhibitor of the enzyme CPT1 involved in FAO, causes ATP depletion and energetic stress, retarding tumor growth in a xenograft model^74^. As FAO activates oncogenic Src and its target genes, as discussed previously, ETX also reverses the Src-regulated gene pattern and abolishes the metastasis potential of TNBC^76^. We have identified that zoledronic acid (ZA), a marketed drug for the management of osteoporosis or bone metastasis, is the direct inhibitor of UGT8 in the sulfatide biosynthetic pathway. Significantly, ZA impedes sulfatide-induced oncogenic signaling, suppressing tumor aggressiveness and pulmonary metastasis of TNBC^112^. In addition, our early work also showed that epalrestat, which is used in the targeted treatment of diabetic complications, inhibits the activity of AKR1B1 and controls the NF-κB pathway, thus attenuating the BCSC phenotype and tumor metastasis^88^ (**Figure 2**). In response to genotoxic chemotherapy exposure, TNBC cells adaptively upregulate pyrimidine synthesis to increase the production of nucleotides necessary for DNA repair, whereas using leflunomide, a clinically approved inhibitor of *de novo* pyrimidine synthesis for the management of rheumatoid arthritis, sensitizes TNBC cells to chemotherapy and causes significant tumor regression when used in combination with doxorubicin^115^. Moreover, leflunomide induces the depletion of nucleotides and the accumulation of DNA damage during replication, leading to synthetic lethality in TNBC cells with the loss of PTEN, which is vital for DNA repair activity^116^. A novel inhibitor of the HBP enzyme PGM3, FR054, reduces both N- and O-glycosylation levels to regulate cell adhesion and migration, impairing tumor growth *in vivo*^114^.

## Concluding remarks and future perspectives

In this review, we outline the metabolic rewiring of glycolysis, OXPHOS, amino acid metabolism, lipid metabolism, and other important branched pathways in TNBC. Cancer cells flexibly regulate key enzymes in these pathways to meet their bioenergetic and biosynthetic demands; subsequent alternations in intracellular energy, biomass precursors, and ROS levels further influence oncogenic signaling, cell viability, proliferation, and CSC phenotype^91^. Understanding the metabolic remodeling mechanisms should help in discovering new metabolic strategies and repurposing existing drugs for the effective intervention of TNBC.

However, metabolic heterogeneity and the adaptive response of TNBC pose considerable challenges to the transition of metabolically targeted therapy to the clinic. Although TNBC is characterized by the metabolic rewiring from OXPHOS to glycolysis, in a specific malignant mass, not all TNBC cells depend on aerobic glycolysis. Indeed, they can also transit to an epithelial-like state to rely on OXPHOS^102^ or stably maintain a hybrid metabolic phenotype with a high activity of both glycolysis and OXPHOS^110^. This metabolic heterogeneity and plasticity of cancer cells arise from inherent gene dysregulation or from extrinsic cues in the tumor microenvironment, such as hypoxia, acidification, nutrient availability, and antitumor drugs. The tumor microenvironment and the different cells in this microenvironment are often modified by the dysregulated metabolism of tumor cells to further facilitate tumor growth in a symbiotic manner^131–133^. Dysbiosis of the microbiome and microbial metabolome also plays a role in breast cancer metabolism and progression^134^. In addition, TNBC itself is a heterogeneous disease that can be classified into four distinct molecular subtypes according to gene expression profiling, with each subtype displaying a differential response to chemotherapy^135^. Therefore, in the future, systemic metabolic therapeutics in combination with radiation, chemotherapy, targeted therapy, or immunotherapy may be used to combat tumor heterogeneity and induce synthetic lethality, ultimately improving outcomes for patients with TNBC.
